# Socioeconomic and Racial Disparities in Cancer Risk from Air Toxics in Maryland

**DOI:** 10.1289/ehp.7609

**Published:** 2005-03-14

**Authors:** Benjamin J. Apelberg, Timothy J. Buckley, Ronald H. White

**Affiliations:** ^1^Department of Epidemiology,; ^2^Department of Environmental Health Sciences, and; ^3^Risk Sciences and Public Policy Institute, Johns Hopkins Bloomberg School of Public Health, Baltimore, Maryland, USA

**Keywords:** air toxics, cancer, disparity, environmental justice, exposure, income, NATA, race

## Abstract

We linked risk estimates from the U.S. Environmental Protection Agency’s National Air Toxics Assessment (NATA) to racial and socioeconomic characteristics of census tracts in Maryland (2000 Census) to evaluate disparities in estimated cancer risk from exposure to air toxics by emission source category. In Maryland, the average estimated cancer risk across census tracts was highest from on-road sources (50% of total risk from nonbackground sources), followed by nonroad (25%), area (23%), and major sources (< 1%). Census tracts in the highest quartile defined by the fraction of African-American residents were three times more likely to be high risk (> 90th percentile of risk) than those in the lowest quartile (95% confidence interval, 2.0–5.0). Conversely, risk decreased as the proportion of whites increased (*p* < 0.001). Census tracts in the lowest quartile of socioeconomic position, as measured by various indicators, were 10–100 times more likely to be high risk than those in the highest quartile. We observed substantial risk disparities for on-road, area, and nonroad sources by socioeconomic measure and on-road and area sources by race. There was considerably less evidence of risk disparities from major source emissions. We found a statistically significant interaction between race and income, suggesting a stronger relationship between race and risk at lower incomes. This research demonstrates the utility of NATA for assessing regional environmental justice, identifies an environmental justice concern in Maryland, and suggests that on-road sources may be appropriate targets for policies intended to reduce the disproportionate environmental health burden among economically disadvantaged and minority populations.

Environmental justice is a term used to describe the movement concerned with inequities in the distribution of adverse environmental and health consequences of industrial activities and environmental policies [U.S. Environmental Protection Agency (EPA) 2004a]. The movement grew from early observations that a seemingly unequal burden of pollution fell on disenfranchised and disadvantaged communities, often characterized by lower incomes and high proportions of minorities (Brown 1995). With the issuance of Presidential Executive Order 12898 in 1994, achieving “environmental justice” was integrated into the missions of all federal agencies (Clinton 1994). The U.S. EPA defines environmental justice to mean that “no group of people, including a racial, ethnic, or a socioeconomic group” should be disproportionately affected by “industrial, municipal, and commercial operations or the execution of federal, state, local, and tribal programs and policies” (U.S. EPA 2004a).

There is ample evidence that minority and low-income communities bear a disproportionate burden of exposure to many environmental contaminants (Brown 1995; Institute of Medicine 1999), including air pollution (Samet et al. 2001; Schweitzer and Valenzuela 2004). The availability of nationwide ambient monitoring for the criteria air pollutants (carbon monoxide, lead, nitrogen dioxide, ozone, particulate matter, and sulfur dioxide) makes assessment of exposure and risk in disadvantaged and minority communities particularly feasible. However, considerably less is known about the distribution of exposure to and risk from the wide range of hazardous air pollutants (HAPs; also known as “air toxics”) identified by Congress in the Clean Air Act Amendments (1990), because nationwide ambient monitoring is not possible because of the sheer number of pollutants and their diverse chemical properties (Caldwell et al. 1998; Morello-Frosch et al. 2000; Woodruff et al. 1998).

In the early 1990s, the U.S. EPA undertook the Cumulative Exposure Project (CEP) with the goal of modeling annual ambient air concentrations of 148 air toxics and their associated risk (Rosenbaum et al. 1999; Woodruff et al. 1998). A recent analysis of modeled national estimates suggests that ambient concentrations of HAPs exceed benchmark risk levels for cancer and non-cancer end points in many areas of the country (Caldwell et al. 1998; Woodruff et al. 1998, 2000). Furthermore, several recent studies have documented a disproportionate burden of air toxics exposure and/or risk falling on minority and low-income populations. These studies have included varying sources of exposure, including high traffic density (Green et al. 2004; Gunier et al. 2003), location of Toxics Release Inventory (TRI) and other treatment, storage, and disposal facilities (Morello-Frosch et al. 2002; Pastor et al. 2001; Perlin et al. 2001), and modeled estimates from the U.S. EPA’s CEP (Lopez 2002; Morello-Frosch et al. 2002). Although these results suggest that mobile sources and large point sources are likely contributors to exposure disparities, none of these studies examined the relative contribution of different source categories in a particular region to estimated risk disparities.

To address this data gap, we examined the U.S. EPA’s 1996 National Air Toxics Assessment (NATA) (U.S. EPA 2002a) in Maryland along with U.S. Census 2000 data (Maryland Department of Planning 2004) to describe the relationship between tract-level socioeconomic and racial characteristics and estimated cancer risk from exposure to air toxics. Because the NATA estimates are source specific, we are able to examine the emission source(s) driving risk disparities and, for socioeconomic characteristics, the sensitivity of this relationship to the measure used to define socioeconomic position. We use Maryland as a case study because of the high cancer rates in the state compared with national averages. For 2000, Maryland’s rate of 48.6 per 10,000 was significantly higher than the national average of 47.3 per 10,000 (Maryland Department of Health and Mental Hygiene 2003). In addition to the elevated cancer rates observed, Maryland ranked 12th among all states in estimated mean risk from cancer-causing air pollutants, based on the U.S. EPA’s 1996 NATA estimates (U.S. EPA 2002a). In this analysis, we investigate whether this apparent excess cancer risk falls disproportionately on economically disadvantaged and/or minority communities, and whether particular sources are primarily associated with these health risks and should be targeted for emissions reductions to help achieve environmental justice.

## Materials and Methods

We examined whether racial and economic disparities in estimated cancer risk from air toxics exist in the state of Maryland, and whether such disparities arise from particular emission source categories. To do so, we obtained modeled cancer risk estimates from the U.S. EPA’s NATA (U.S. EPA 2002a) and linked them to socioeconomic and racial characteristics from the 2000 U.S. Census (Maryland Department of Planning 2004) for all census tracts in the state of Maryland. We chose the census tract as the unit of analysis to examine the relationship between a community’s economic and racial makeup and risk from exposure to air toxics. Further, the tract is the smallest unit for which estimated cancer risks are available.

### U.S. EPA’s NATA.

The NATA and its predecessor the CEP provide an established means for using source emission data to derive estimates of ambient air toxin exposure (Rosenbaum et al. 1999) and its associated cancer risk (Caldwell et al. 1998; Woodruff et al. 1998, 2000). We downloaded the NATA cancer risk estimates at the census tract level (U.S. EPA 2002a) and extracted results for Maryland. The U.S. EPA’s most recent national-scale air toxics assessment was conducted for 1996 and estimates the annual aggregate cancer risk for 29 chemicals (U.S. EPA 2004b). The methods used to generate census tract–level estimates of risk are described in detail by the U.S. EPA (U.S. EPA 2004b). In brief, NATA combines source emission data (i.e., TRI data, databases from the U.S. EPA’s Maximum Achievable Control Technology program, and emissions estimates for mobile and area sources) with meteorology (wind speed and direction) in a Gaussian dispersion model [Assessment System for Population Exposure Nationwide (ASPEN)] that accounts for atmospheric decay to provide an estimate of the annual ambient air toxin concentration (U.S. EPA 2003). Estimates of ambient concentrations from ASPEN are then included in an inhalation model called the Hazardous Air Pollution Exposure Model 4 (HAPEM4). This model incorporates activity patterns that may influence personal exposure to ambient pollutants.

From these concentration estimates, NATA further estimates cancer risk by applying inhalation unit risk factors according to U.S. EPA standard methods (U.S. EPA 1992 U.S. EPA 2004b). For cancer, even though the type (e.g., liver, blood, lung) and weight of evidence (e.g., known, suspected, or possible) varied by chemical, aggregate risk was estimated as the sum of individual chemical risks. The cancer risk estimates are considered by the U.S. EPA to be “upper-bound” estimates—“a plausible upper limit to the true probability that an individual will contract cancer over a 70 year lifetime as a result of a given hazard (such as exposure to a toxic chemical)” (U.S. EPA 2002c).

The following emission source categories are included in the inventory and subsequent assessment (U.S. EPA 2002c): *a*) Major emissions sources were “stationary facilities that emit or have the potential to emit 10 tons of any one toxic air pollutant or 25 tons of more than one toxic air pollutant per year” (e.g., electric utility power plants, oil refineries). *b*) Area and other emissions sources were “sources that generally have smaller emissions on an individual basis than ‘major sources’ and are often too small or ubiquitous in nature to be inventoried as individual sources”; this may include smaller facilities (e.g., dry cleaning facilities, gas station/automobile repair) or other sources such as wildfires. *c*) On-road mobile sources were “vehicles found on roads or highways,” and *d*) nonroad mobile sources were “mobile sources not found on roads and highways (e.g., airplanes, trains, lawn mowers, construction vehicles, farm machinery).” In addition, background concentrations are estimated, which represent exposure from “natural sources, persistence in the environment of past years’ emissions and long-range transport from distant sources.”

### Linking NATA risk estimates with census data.

We obtained U.S. Census 2000 data for the state of Maryland from the Maryland State Data Center (Maryland Department of Planning 2004). The choice of socioeconomic measures was guided by Krieger et al. (1997) and encompasses indicators of income, wealth, poverty, and education. In particular, we extracted the following year 2000 census tract level data: median household income in 1999 (US$), per capita income in 1999 (US$), percentage of households owner occupied, percentage of households with public assistance income for 1999, percentage living below the poverty level in 1999, and percentage of the population ≥25 years of age without a high school diploma. Additionally, we examined the percentage of the population composed of whites, African Americans, and Hispanics, where the percentages are based on those who consider themselves “white only” or “African-American only.”

NATA cancer risk estimates were calculated for the year 1996 and use 1990 census tracts. In the 2000 Census, several changes were made to census tract boundaries. The U.S. Census Bureau provides a set of census tract relationship files that link the 1990 and 2000 census tracts (U.S. Census Bureau 2003). We downloaded this file for Maryland, which contains the proportion of the population in a given year 2000 census tract coming from redefined 1990 census tracts.

To link NATA risk estimates among 1990 census tracts with 2000 census tracts, we identified the NATA cancer risk estimates for the 1990 census tracts and constructed weighted averages of risk for the 2000 census tracts, based on the 2000 population proportions as follows:


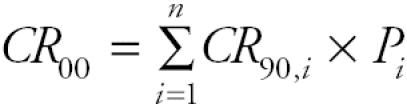


*CR*_00_ is the cancer risk in the year 2000 census tract, *CR*_90_*_,i_* is the cancer risk in the year 1990 census tract *i*, *P_i_* is the proportion of the 2000 census tract population coming from 1990 census tract *i*, and *n* is the number of 1990 census tracts at least partially contained in the 2000 census tract. This calculation was performed for all source categories (total, major, area, on-road, nonroad, and background).

### Statistical analysis.

We downloaded NATA data (U.S. EPA 2002a) and racial/socioeconomic data from the U.S. Census 2000 (Maryland Department of Planning 2004) as Excel spreadsheets and the census relationship file for Maryland (U.S. Census Bureau 2003) as a text file, which we imported into Excel. Data linking and data management were performed in SAS (SAS Institute Inc., Cary, NC), and statistical analyses were performed in STATA (StataCorp, College Station, TX). We initially treated cancer risk as a continuous variable and explored the relationships between median household income, per capita income, and race and tract-level cancer risk estimates. We used a linear regression model to estimate the average change in estimated cancer risk associated with changes in income and racial distribution. The Breusch-Pagan/Cook-Weisberg test was used to identify the presence of heteroskedasticity (“hettest” in STATA 8.0), in which case robust SEs were used. Multivariate models included race as a linear predictor and income as a quadratic term or indicator variable (quartiles). We also included interaction terms in multivariate models to look for the presence of effect modification between income and race on estimated cancer risk.

We then divided census tracts into quartiles defined by each of the socioeconomic and racial characteristics. We calculated the proportion of census tracts in each quartile that were “high risk,” defined as greater than the 90th percentile of cancer risk among all Maryland tracts. We used Pearson’s chi-square tests to test for differences in proportions across quartiles. We also estimated relative risks (RRs) and 95% confidence intervals (CIs) for being high risk across quartiles of socioeconomic and racial characteristics. This analysis was performed for each of the socioeconomic indicators and race for all source categories.

## Results

Four census tracts in the NATA file, consisting of 88 individuals, were excluded because the corresponding tracts were not present in the 2000 census relationship file (U.S. Census Bureau 2003). Two additional tracts were excluded because they had a population size of zero. Finally, we excluded five tracts whose entire population was housed in “group quarters” because no median household income measure was available and these tracts were not informative with respect to the hypothesis under study.

Table 1 presents the distribution of racial and socioeconomic characteristics among Maryland census tracts in 2000, along with estimated cancer risk from air toxics. Considerable variability exists in the distributions of socioeconomic and most racial indicators among Maryland census tracts. However, little variability was observed for the percentage of Hispanic residents because most tracts had few Hispanics. For example, in 75% of the census tracts, < 4% of the residents identified themselves as Hispanic. The correlation between socioeconomic and racial characteristics is shown in Table 2.

The cancer risk estimates shown in Table 1 were derived from population-weighted averages of the 1996 NATA estimates, as described above. The average estimated cancer risk from all sources was 5.8 × 10^−5^, suggesting a greater than one in a million lifetime excess cancer risk. In fact, the lowest cancer risk estimate among the census tracts was 2.3 × 10^−5^, 20 times higher than this commonly used regulatory threshold (Clean Air Act Amendments 1990). Among source contributions, on-road sources provide the greatest contribution to cancer risk (on average, 50% of total risk from nonbackground sources), followed by nonroad (25%) and area sources (23%). By comparison, major sources contribute significantly less to the overall cancer risk burden (< 1%).

**Figure 1:**
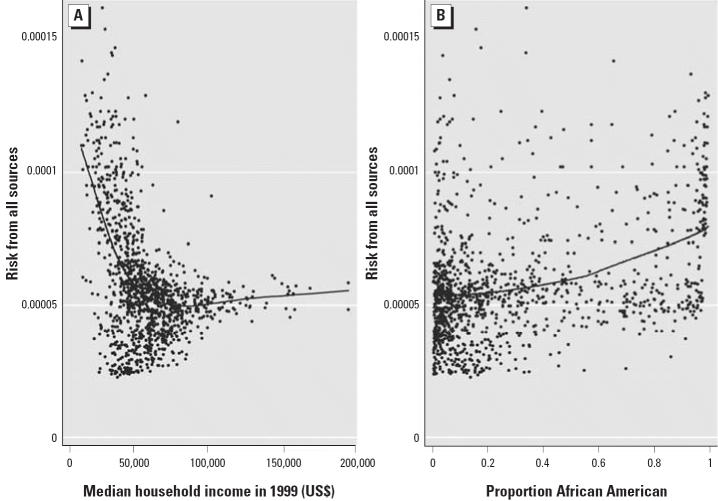
Relationship of estimated cancer risk from air toxics with median household income (*A*) and proportion of African-American residents (*B*) among Maryland census tracts, 2000. Line represents a lowess smoothing function.

We examined the relationships between risk from all sources and household income and per capita income using scatter plots. The trend in risk as a function of income was similar for the two indicators, so only median household income is shown here (Figure 1A). As shown in Figure 1A, the relationship between risk and income differs by level of income. Below a median household income of $50,000, an estimated decrease in risk of 1.2 × 10^−5^ was associated with each $10,000 increase in income (*p* < 0.001). Above $50,000, there was no statistically significant association between median household income and estimated cancer risk at the census tract level (β = 2.9 × 10^−7^ per $10,000; *p* = 0.11). An analysis by race showed an average decrease in estimated cancer risk of 2.6 × 10^−4^ for every 10% increase in the percentage of whites living in a census tract (*p* < 0.001). Conversely, an increase in risk of the same magnitude (2.6 × 10^−4^) was observed for a 10% increase in the percentage of African Americans (*p* < 0.001; Figure 1B). No significant association was observed between Hispanic ethnicity and total risk.

We then examined the relationship between quartiles of the various socioeconomic indicators and race and the probability of a tract being high risk (defined as greater than the 90th percentile of risk; Table 3). If there were no relationship between racial and socioeconomic characteristics and risk, then the proportion of high-risk tracts should be similar among quartiles. We did not find this to be the case. For example, census tracts with the highest proportion of whites were one-third as likely to be high risk compared with the lowest quartile (95% CI, 0.17–0.45). Conversely, tracts in the highest quartile defined by proportion of African Americans were three times as likely to be high risk compared with the lowest quartile (95% CI, 2.0–5.2). Census tracts with higher proportions of Hispanics were less likely to be high risk; however, the small range in the proportion of Hispanics living in a census tract limits interpretation of these results. For this reason, Hispanic ethnicity was not analyzed further.

The disparities observed were even greater when stratifying by income and education levels. For example, census tracts in the lowest quartile of median household income were 100 times more likely to be high risk than were those in the highest quartile (95% CI, 14–715). Furthermore, an increasing trend in the percentage of high-risk tracts was observed from the highest to the lowest quartile of median household income (0.3, 1.0, 5.6, and 33% for the fourth, third, second, and first quartile, respectively). Similar results were observed for other socioeconomic indicators (Table 3), although the magnitude differed by indicator used. For per capita income, the percentage of high-risk tracts increased from 2.6 to 29% from the highest to lowest quartile (RR = 1.0, 2.1, and 11 comparing the third, second, and first quartiles with the fourth). For the remaining indicators, trends in the RR of being high risk were apparent from highest to lowest levels of socioeconomic position (proportion owner occupied: RR = 3.3, 14, 22; proportion below poverty: RR = 2.0, 18, and 100; proportion without a high school diploma: RR = 1.0, 4.0, and 34; proportion with public assistance income: RR = 0.7, 3.3, and 15).

**Figure 2:**
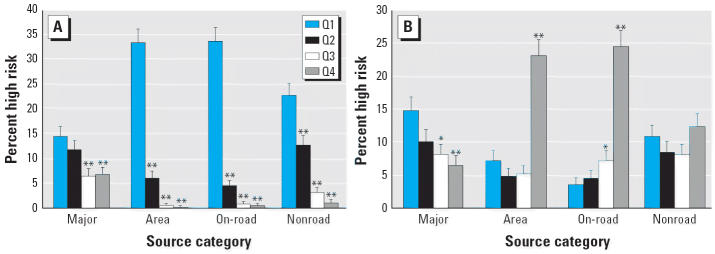
Percentage of high-risk tracts by emission source category and quartile (Q) of median household income (*A*) and proportion African-American residents (*B*). High risk is defined > the 90th percentile of risk from each source among Maryland census tracts. Error bars represent SE.
*p < 0.05;
**p < 0.01 using Pearson’s chi-square test to compare each quartile within a source category to the first.

An examination of socioeconomic disparities in cancer risk by emission source category revealed significant disparities for on-road, area, and nonroad sources. Given the correlation between different socioeconomic indicators (Table 2), we focus here on the results for median household income. Figure 2A shows the percentage of census tracts defined as high risk from each source category by quartile of median household income. For on-road, area, and nonroad sources, census tracts in the lowest quartile of median household income were 51 (95% CI, 13–206), 101 (95% CI, 14–722), and 17 (95% CI, 6.4–47) times more likely than the highest quartile to be high risk. Furthermore, the proportion of high-risk tracts monotonically decreased with increasing income. Similar trends were observed when using other socioeconomic indicators, although the magnitude varied. For example, the RRs for highest versus lowest quartiles of per capita income was 8.0 (95% CI, 4.4–15) for on-road sources, 12 (95% CI, 5.7–23) for area sources, and 4.7 (95% CI, 2.7–8.2) for nonroad sources. Comparatively less evidence of a socioeconomic disparity was observed for cancer risk from major sources. For major sources, the magnitude of the difference in cancer risk between the highest and lowest quartiles of the various socioeconomic indicators ranged from 0.9- to 2.8-fold.

Similarly, the strongest racial disparities in estimated cancer risk were observed among on-road and area sources. Figure 2B shows the percentage of high-risk census tracts from each source category by quartile of proportion of African Americans in the population. Significant differences in the proportions were observed for on-road (RR = 6.2; 95% CI, 3.5–11 comparing highest with lowest quartile) and area sources (RR = 3.0; 95% CI, 2.0–4.7 comparing highest with lowest quartile). In contrast, for major sources, a statistically significant reduction in the proportion of high-risk tracts was observed as the proportion of African Americans residing in a census tract increased. Opposite effects were observed for quartiles defined by the proportion of white residents (data not shown). Finally, we oberved no significant differences among quartiles defined by the proportion of white residents for risk from nonroad sources.

**Figure 3:**
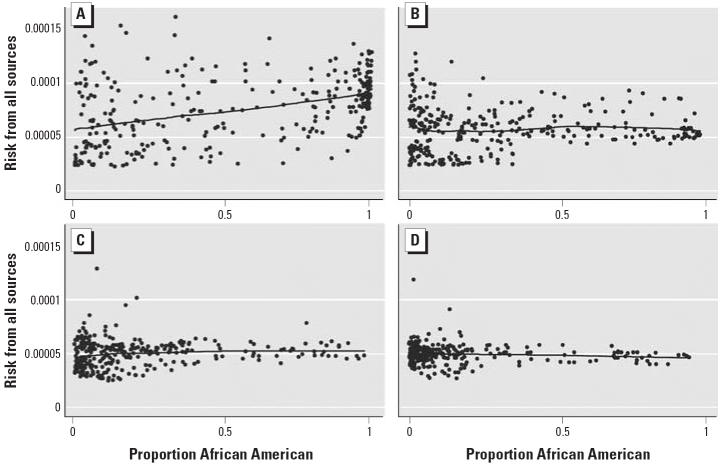
Risk from all sources as a function of the proportion of African-American residents in the first (*A*, lowest), second (*B*), third (*C*), and fourth (*D*, highest) quartiles of median household income. Line represents a lowess smoothing function.

To examine the joint effects of race and income on estimated cancer risk, we ran a linear regression model, with interaction terms, of estimated cancer risk on median household income and percentage of African Americans. We found evidence of an interaction between the effects of income and race on risk (*p* < 0.001). Specifically, the strongest association between race and risk was observed in the lowest quartile of median household income (Figure 3A). In this quartile, a 10% increase in the percentage of African Americans in the tract was associated with an average increase in risk of 3.4 × 10^−4^. By contrast, in the highest quartile of income (Figure 3D), we observed a slight but statistically significant reduction in risk with increasing percentage of African Americans. Because the strongest disparities in cancer risk were observed from area and on-road sources, we performed a similar analysis using estimated risk from these sources. Once again, interaction terms were statistically significant (*p* < 0.001), with a stronger effect of race on risk at lower incomes.

## Discussion

In this analysis, we characterized the relationship between estimated cancer risk from air toxics and socioeconomic and racial characteristics at the census tract level in Maryland. We found strong and consistent associations between socioeconomic and racial characteristics of census tracts and estimated cancer risk from air toxics. Census tracts were more likely to be characterized as high risk as the level of socioeconomic disadvantage (as measured by several indicators) increased, the proportion of white residents decreased, and the proportion of African-American residents increased. In general, risk declined as the proportion of Hispanic residents increased; however, there were relatively few tracts with a large proportion of Hispanic residents. Although income, education, and race were all significantly associated with estimated cancer risk, the magnitude of disparities observed was more pronounced for income and education compared with race.

Economic and racial disparities in estimated cancer risk were not uniformly observed for all emission source categories. Significant disparities among tracts defined by income and education level were observed for area, on-road, and nonroad sources. For these sources, census tracts in the lowest quartiles of median household income were 15- to 100-fold more likely to be high risk than those in the highest quartile of income. For tracts defined by racial distribution, statistically significant disparities were observed only for area and on-road sources. Conversely, risk from major sources was more evenly distributed among census tracts defined by income and education. In contrast to the other source categories, for major sources, census tracts with an increasing fraction of whites and a decreasing fraction of African-American residents yielded an increased risk. However, because high risk was defined as the top 10% of risk and major sources were a small contribution to overall risk, the impact of this association may have minimal public health relevance.

In a recent analysis of results from the U.S. EPA’s CEP, Morello-Frosch et al. (2002) reported that mobile sources drive cancer risk from air toxics in southern California, whereas area and point sources are drivers of air toxics exposure. Although we did not examine source contributions to air toxics exposure, our risk findings were consistent; that is, on-road sources were the greatest contributor to cancer risk among census tracts in Maryland, followed by nonroad sources (Table 1). The difference in source contributions to estimated exposure and cancer risk may be due to a lack of cancer potency data for compounds released from point sources, emissions of more potent carcinogenic compounds from mobile sources, and/or a greater likelihood for personal exposure from mobile sources (Morello-Frosch et al. 2000).

In examining race and income concurrently, Morello-Frosch et al. (2001) reported a relatively consistent disparity in population-weighted individual cancer risk between racial/ethnic groups across income strata in southern California. This differs from our results, which use the census tract as the unit of observation. We found little evidence of a disparity in risk, at higher incomes, between tracts with large differences in racial makeup. It is not clear whether the different inferences regarding the joint effects of race and income reflect differences in methodology or variation in source and demographic characteristics between the two study regions.

In our analysis, on-road sources were significantly associated with the racial and socioeconomic characteristics of census tracts in Maryland. The finding of a potential disparity in cancer risk from on-road sources is not surprising, given the likelihood for poorer neighborhoods to be in the midst of high-traffic congested areas. Gunier et al. (2003) studied the relationship between traffic density and socioeconomic level and race in California. They found that the census block groups in the lowest quartile of median family income were more likely to have high traffic density than were the highest quartile. Furthermore, the inverse relationship between median income and traffic density was observed for all race/ethnicities except whites (Gunier et al. 2003). In another recent study of traffic exposure and public school locations in California, Green et al. (2004) reported that schools located near high-traffic areas were more likely to be “economically disadvantaged” and “nonwhite.” Therefore, the results of this study are supported by a growing body of evidence indicating that low-income and minority populations are more likely to reside and attend school near sources of on-road pollution, and that the relationship between income and exposure may differ by race.

One unexpected finding was the lack of a consistent association between risk from major sources and tract-level income characteristics. Recent studies have documented racial and economic disparities in the location of TRI and other treatment, storage, and/or disposal facilities (Morello-Frosch et al. 2002; Pastor et al. 2001, 2002; Perlin et al. 2001). The potential for long-range transport of air pollutants from major point sources may attenuate any disparities in cancer risk that would be expected on the basis of disparities in the location of treatment, storage, and disposal facilities. It has also been suggested (Morello-Frosch et al. 2002; Pastor et al. 2001) that the relationship between income and exposure from major point sources may have an inverted U-shape. The areas with the lowest income have little exposure because of lack of economic and industrial development, and areas with the highest income have little exposure because of increased mobility and political will. Under this scenario, the burden of exposure would fall on low- to middle-income working-class populations (Morello-Frosch et al. 2002; Pastor et al. 2001). We observed no suggestion of such a U-shaped pattern (data not shown).

There are several limitations to the NATA analysis, some of which reflect inherent limitations in the risk assessment process (U.S. EPA 2002b). The cancer risk assessment was limited to 29 air toxics with sufficient emission and risk estimate data; therefore, the cancer risk estimates are not a comprehensive assessment of all air toxics of concern. As mentioned above, diesel exhaust was excluded from the cancer risk estimation because of the lack of EPA consensus on a cancer risk estimate. This would have implications for overall risk from on-road and nonroad sources and, likely, the magnitude of disparity observed. Further, threshold reporting of emissions from major point source databases such as TRI may have underestimated risk from these sources.

The U.S. EPA’s analysis focuses only on inhalation exposure from air toxics, omitting exposure from other pathways (e.g., dermal and ingestion). To the extent that these other pathways contribute to risk, cancer risk estimates would be underestimated. Furthermore, several studies have reported that modeled and measured outdoor levels of volatile organic compounds (VOCs) underestimate indoor concentrations and personal exposures. A recent study conducted in several south Baltimore communities concluded that personal exposures tend to be higher relative to parallel measurements made indoors and outdoors. For many VOCs, indoor concentrations dominated exposure; however, the authors reported that compounds associated with vehicle emissions were found to have similar indoor and outdoor concentrations (Payne-Sturges et al. 2004). In a similar study in three Minnesota communities, personal exposure to VOCs was consistently higher than indoor and outdoor concentrations (Sexton et al. 2004). Thus, cancer risk estimates based on personal monitoring would likely be higher than those based on estimated outdoor concentrations. However, even with the imprecision in exposure and risk estimates, the NATA results should provide a good indication of the relative levels of source emissions among communities. Results from the present study indicate that HAP source emissions are higher among minority and economically disadvantaged communities.

An additional source of uncertainty arises from the comparison of 1996 risk estimates to racial and socioeconomic measures from 2000 census tracts. Significant emission reductions have taken place since the mid-1990s as a result of federal, state, and local efforts, thereby affecting the magnitude of cancer risk (U.S. EPA 2002b). It is unlikely, however, that significant changes in all of the socioeconomic measures evaluated would have occurred in such a short time frame, so this analysis can be seen as an estimate of the relationship between racial and socioeconomic characteristics and estimated cancer risk from air toxics as of the mid-1990s. Furthermore, for on-road mobile sources, it is likely that the observed risk and disparity have increased in proportion to increases in vehicle miles traveled and the proportion of less fuel-efficient sports utility vehicles (de Abrantes et al. 2004; Schmitz et al. 2000; U.S. EPA 2000).

In conclusion, these results provide evidence that cancer risk associated with air toxics exposure, particularly from on-road and area sources, disproportionately falls onto socioeconomically disadvantaged and African-American communities. This research also highlights the potential for confounding by socioeconomic status when examining the long-term health effects of traffic-related pollutants, because lower socioeconomic status is associated with a host of adverse health effects that may or may not be mediated through the effects of air pollution. Additional analyses should be performed nationwide to examine whether similar relationships exist across different regions of the country and which compounds are the primary determinants of this risk disparity. Furthermore, future research should explore the complex interactions between race and income on risk from air toxics exposure. In the interim, these data, along with prior literature on the health effects associated with residing in close proximity to high traffic density, suggest that efforts to reduce the disproportional health risk burden falling on lower income and minority populations should include policies targeting emissions from on-road vehicle sources.

## Figures and Tables

**Table 1:** Distribution of demographic characteristics and estimated cancer risk from air toxics among Maryland census tracts, 2000 (*n* = 1,210 tracts).

		Percentile				
Characteristic	Mean	5th	25th	50th	75th	95th
Median household income (US$)*^a^*	55,010	21,188	38,577	50,910	67,339	99,531
Per capita income (US$)*^a^*	24,952	11,679	18,135	23,247	28,671	45,589
Percent owner-occupied homes	68	20	52	74	87	95
Percent with public assistance income*^a^*	2.8	0	0.7	1.6	3.2	11
Percent below poverty level*^a^*	10	1.4	3.4	6.3	13	33
Percent without a high school diploma	18	3.3	9.1	16	25	42
Percent white	63	1.9	38	75	90	97
Percent African American	30	0.8	4.4	15	47	96
Percent Hispanic	4.1	0.5	1.1	1.8	3.7	17
Cancer risk						
All sources*^b^*	5.8 × 10^−5^	2.8 × 10^−5^	4.4 × 10^−5^	5.3 × 10^−5^	6.5 × 10^−5^	1.1 × 10^−4^
Major sources	2.8 × 10^−7^	4.0 × 10^−8^	1.5 × 10^−7^	2.7 × 10^−7^	3.4 × 10^−7^	5.8 × 10^−7^
Area sources	8.8 × 10^−6^	2.4 × 10^−6^	5.2 × 10^−6^	7.2 × 10^−6^	1.0 × 10^−5^	2.2 × 10^−5^
On-road sources	1.9 × 10^−5^	3.5 × 10^−6^	1.2 × 10^−5^	1.6 × 10^−5^	2.2 × 10^−5^	4.5 × 10^−5^
Nonroad sources	9.6 × 10^−6^	1.6 × 10^−6^	6.2 × 10^−6^	9.3 × 10^−6^	1.2 × 10^−5^	2.0 × 10^−5^

a Estimates are for 1999.

b Includes background sources.

**Table 2:** Correlation between census tract level demographic characteristics in Maryland, 2000 (*n* = 1,210 tracts).

	Percent white	Percent African American	Percent Hispanic	Median household income	Per capita income	Percent owner occupied	Percent below poverty level	Percent with public assistance	Percent without high school diploma
Percent white	1.0000								
Percent African American	−0.9705	1.0000							
Percent Hispanic	−0.1632	−0.0300	1.0000						
Median household income	0.3449	−0.3909	0.0089	1.0000					
Per capita income	0.3879	−0.4241	−0.0391	0.8568	1.0000				
Percent owner occupied	0.4820	−0.4413	−0.2209	0.6210	0.4470	1.0000			
Percent below poverty level	−0.4698	0.4954	−0.0170	−0.6240	−0.5400	−0.6061	1.0000		
Percent with public assistance	−0.5167	0.5612	−0.0781	−0.5391	−0.4859	−0.4778	0.7866	1.0000	
Percent without high school diploma	−0.3626	0.4013	0.1001	−0.6880	−0.6731	−0.4192	0.7023	0.6889	1.0000

**Table 3:** Percentage of high-risk tracts and RRs by quartile of demographic measure in Maryland, 2000.

Census tract measure	Percent high risk^a^	RR (95% CI)
Median household income		
Quartile 1	33	100 (14–715)
Quartile 2	5.6	17 (2.3–127)
Quartile 3	1.0	3.0 (0.3–29)
Quartile 4	0.3	—
Per capita income		
Quartile 1	29	11 (5.5–22)
Quartile 2	5.6	2.1 (0.9–4.9)
Quartile 3	2.7	1.0 (0.4–2.6)
Quartile 4	2.6	—
Percent owner occupied		
Quartile 1	22	22 (6.9–68)
Quartile 2	14	14 (4.5–46)
Quartile 3	3.3	3.3 (0.9–12)
Quartile 4	1.0	—
Percent with public assistance income		
Quartile 1	2.0	—
Quartile 2	1.3	0.7 (0.2–2.3)
Quartile 3	6.6	3.3 (1.4–8.2)
Quartile 4	30	15 (6.7–34)
Percent below poverty level		
Quartile 1	0.3	—
Quartile 2	0.7	2.0 (0.2–22)
Quartile 3	6.0	18 (2.4–134)
Quartile 4	33	100 (14–710)
Percent age ≥ 25 without a high school diploma		
Quartile 1	1.0	—
Quartile 2	1.0	1.0 (0.2–4.9)
Quartile 3	4.0	4.0 (1.1–14)
Quartile 4	34	34 (11–107)
Percent white		
Quartile 1	22	—
Quartile 2	6.9	0.32 (0.20–0.51)
Quartile 3	5.6	0.26 (0.16–0.44)
Quartile 4	5.9	0.28 (0.17–0.45)
Percent African American		
Quartile 1	6.6	—
Quartile 2	5.3	0.8 (0.4–1.5)
Quartile 3	6.6	1.0 (0.6–1.8)
Quartile 4	22	3.2 (2.0–5.2)
Percent Hispanic		
Quartile 1	17	—
Quartile 2	8.3	0.48 (0.31–0.75)
Quartile 3	8.0	0.46 (0.29–0.73)
Quartile 4	6.6	0.38 (0.23–0.63)

Quartile 1 is lowest; quartile 4 is highest.

a > the 90th percentile of risk among Maryland census tracts.
